# Principles of Human Physiology; with Their Chief Applications to Pathology, Hygiene, and Forensic Medicine

**Published:** 1850-02

**Authors:** 


Art. V.—*Principles of Human Physiology; with their Chief Applications to Pathology, Hygeine, and Forensic Medicine.* By William B. Carpenter, M. D., F. R. S., F. G. S., Examiner in Physiology in the University of London; Lecturer on Physiology at the London Hospital Medical School. Fourth American Edition. With extensive additions and improvements, by the author. With two plates and three hundred and four wood cuts. Philadelphia: Lea & Blanchard. 1850.

The unresting, unappeaseable inquiries of the mind in the science of physiology are making marked and decided progression. The man who may have known all that had been revealed forty or fifty years ago, and who is content to rest upon the acquisitions of that period, would, if he were to appear in an assembly of modern physiologists, be justly considered a fossil remain of science, and might excite no little inquiry as to the precise period of the eocene formation to which he might belong. If a pterodactyl were to flit around an assemblage of geologists, or an icthyosaurus were to walk into one of their meetings, the appearance of two such wonders would excite scarcely any more surprise, than the utterances of an ancient physiologist would among the modern cultivators of the science of life. Science has extended her domain so greatly, that she can now afford to be rigid in her demands. She asks for facts, not speculation; she seeks truth, and will not accept, in lieu of it, fine-spun theory, piebald declamation, jejune rigmarole, nor arrogant dogmatism. She holds no communion with him who blindly embraces solidism, and shows his devotion to truth by dexterously avoiding every tint and shade of humoralism. Nor does she any more readily extend her hand to him, who leaps into the depths of humoralism, and exhibits his love of truth only in a hatred of everything like solidism. The votaries of science must follow where she leads, and he who enters upon the paths that run through her domain, must content himself to reach her temples, and drink of her fountains upon her own terms. The days of Whytte and Haller have gone; a new creation has dawned upon physiological science, and the new heavens and the new earth must be surveyed by the light that is "set in the new firmament." By this course alone can any man hope to count evenings and mornings as days.

When Cuvier first blazed a path through the regions of the animal kingdom, physiology began to awaken from the slumbers and darkness that had bound it. Inquirers rushed into the field, and light began to shine in the midst of the deepest gloom. All the phenomena of life were diligently observed, and as far down in the scale of living beings, that were open to the natural eye, as it was possible to go, the physiologist went. When this world had been thoroughly explored and conquered, the microscope was called into the field, and myriads of forms, unknown to natural vision, rose at once to view, and developed to the wonder-stricken mind a new world of interminable interest. When the mind sounded the profound depths of life, revealed by the microscope, and, linking vegetable with animal physiology, traced vitality from its manifestations in the humblest monad, through every grade of organism up to its perfection in man; when it looked narrowly into every form of life, and patiently, wisely, and carefully gathered facts from Nature's vast storehouses, physiology began for the first time to assume the dignity and character of a science. The worn-out tissues of a bye-gone age were rolled up as an old garment, and delivered over to the things that belong to decay.

What a world of enlightenment is open to the diligent and careful microscopist! The microscope is fast rising into a necessity to the perfection of the physician. It is becoming essential to the anatomist, physiologist, and pathologist, and is destined to play an important part in the investigations of each. We shall not now insist upon the importance of this instrument, for its development requires more space than we can now devote to it, but this much we can say, the use of the microscope will amply repay any cultivated mind in the enjoyment afforded by an examination of the world of singular beauty revealed by that instrument, independently of any practical benefit that may be derived from it. A philosophical and keen-sighted poet has said with truth,

"A thing of beauty is a joy forever,"

and the microscopist will feel this truth constantly rising before him, as the infusorial and zoophytic world display their wondrous forms upon the field of vision in his instrument. A cultivated and tasteful mind might feast for weeks upon rotophers and vorticellæ alone, but these constitute but a minute portion of the joys in store for the microscopist. There is an education of the eye for the use of this instrument, which a physician should speedily endeavor to acquire. He cannot too soon undertake to investigate all the phenomena of diseased action that can be brought within the compass of the microscope, and we feel that we are on safe ground in saying that diagnosis is ultimately to receive no ordinary degree of accuracy from the revelations of the comparatively new instrument of which we speak. Of one thing we feel very sure; it is impossible to teach anatomy or physiology successfully without the use of the microscope, and it is very difficult for the student to acquire either, without it. It would be as easy to teach the multiplication table without the digits, as to impart the elementary truths of physiology without the microscope.

The work before us owes much of its excellence to the researches of the microscope, and we feel it incumbent upon us to urge the importance of this book upon the attention of medical readers. A good deal of the present edition has been prepared expressly for the American publishers. Dr. Carpenter says: "The principal changes will be found in the portion of the work which treats of the nervous system; and in the chapter on generation.—The former has been rendered necessary by the progress of the author's own inquiries, which have led him to relinquish certain parts of Dr. Marshall Hall's doctrines, long advocated by himself, and to substitute what he believes will be found a far simpler and more consistent view of the constitution of the cerebro-spinal centres, which may be said to be essentially based on the doctrines of Messrs. Todd and Bowman, though differing from them in some important particulars. * * * * The alterations in the chapter on generation, have chiefly consisted in the substitution of the views of Bischoff, with respect to the development of the ovum, for those of Dr. Barry; the author having satisfied himself that the latter are no longer tenable, whilst the statements of the former, though imperfect in many important particulars, are, on the whole, deserving of the credit which he had previously accorded to Dr. Barry's observations."

"Numerous other additions and alterations, which, taken collectively, constitute no inconsiderable amount of matter, are scattered through the treatise; their importance being greatest, however, in the chapter on the primary tissues, and in that on nutrition."

These quotations will serve to indicate the important character of the changes to be found in this fourth American edition. These changes contribute greatly to the perfection of the work, and make it one of the most complete works of the kind before the American public. The chapters devoted to an examination "of the place of man in the scale of being," "of the elementary parts of the human fabric;" in the latter of which, the whole cell system is most lucidly and thoroughly expounded—"the functions of the nervous system," "on nutrition," and "on generation," are models of philosophical research and analysis.

There is an error on the subject of superfetation, which we may as well correct. Dr. Carpenter says: "In the slave States of America, *it is not uncommon* for a black woman to bear at the same time a black and a mulatto child; the former being the offspring of her black husband, and the latter of her white paramour." Now we think this is rather an uncommon event in the slave States of America. It occurs more frequently than the creation of a world, but not as often as a Philadelphia riot.

In the appendix, the reader will find in the remarks on phrenology, some evidence of that unresting, unappeasable spirit of inquiry, to which we have referred. Some years ago, the learning, eloquence, abilities, energy and zeal of Gall and Spurzheim introduced a system which was called phrenology. The two illustrious Germans travelled over the earth, making converts wherever they went. The system of phrenology stood, apparently, on impregnable grounds, and it grew quite as much through the weakness of its opponents, as by the strength of its advocates. Observation and partial examinations of comparative anatomy seemed to give character and coherence to the views promulged by Gall and Spurzheim, and in the then existing state of knowledge, it was found impossible to find facts subversive of the fundamental truths of the new science.

But it is now evident that the claims of Phrenology cannot be reconciled with an extended knowledge of comparative anatomy, and we are compelled to make great modifications in what was formerly considered beyond the reach of cavil. Dr. Carpenter expresses himself on these subjects in a very becoming spirit, and we commend his remarks to the attention of our readers. He says: "his objections to the *present system* of phrenology are based only on the very imperfect manner in which it has been constructed; the foundations on which it rests being (in his opinion) of a very insecure character; and the superstructure having been built up of the slightest possible material."

"The present system of phrenology is altogether inconsistent with well ascertained facts, regarding the comparative anatomy and embryological development of the cerebrum. It is clearly established by anatomical research, that the *posterior* lobes of the cerebrum are relatively much smaller in the quadrumana than they are in man, and that they disappear altogether in the carnivora, not a vestige of them being discoverable in any of the lower mammalia; and that the *middle* lobes, though they may be traced in the lowest of the mammalian class, are altogether wanting in birds, reptiles, and fishes. The cerebrum of these animals, therefore, is the rudiment of the *anterior* lobe only of that of mammalia. Further, it has been lately shown by Prof. Retzius (whose researches on this head are confirmatory of those of Tiedmann, at the same time being more full and precise), that the development of the cerebrum of the human embryo takes place on the same plan. * * * * * The exact mutual confirmation afforded by these two sources of knowledge, proves the complete inadmissibility of the ordinary phrenological interpretation of the increased development of the posterior lobes in man,—namely, that they are present in all vertebrata, but that they are pushed backwards in the higher forms by the increased development of the anterior portion of the cerebrum. Now, as the instincts and propensities are located, according to the present system of phrenology, in the posterior and middle lobes of the cerebrum, which are altogether wanting in the oviparous classes, in which these instincts and propensities most strikingly manifest themselves, it would appear that some fundamental error must exist in the allocation."

Passing over the series of objections, under the third head, we come to those contained under the fourth. Dr. Carpenter says: "The present system of phrenology leaves undetermined a very large proportion of the cerebral surface in man,—probably not less than one-half; namely, the whole series of convolutions covering the opposing median surfaces of the hemispheres, the convolutions of nearly the whole of the base of the cerebrum, and those of the fissure of Sylvius. Yet not the slightest ground can be adduced for the supposition that these unappropriated portions have any less participation in the operations of the intellect, the exercise of the moral feelings, or the influence of the animal propensities, than have the external and superior portions of the respective lobes. No admission of the imperfection of the present system of phrenology can be a sufficient explanation of this fact, which would seem to indicate some fundamental error in the method employed; since one of the great claims which is set up in behalf of that system is the completeness of the system of psychical philosophy which it presents; so that if there were every facility for observing the relative development of the parts of the cerebral surface in question, there would seem to be no "organs left to distribute over it."

These objections are fatal to the present system of phrenology, but not, in our judgement to the fact that the brain is the organ of the mind.

We commend Dr. Carpenter's work to the serious study of our readers. It is full of invaluable instruction, and deserves the closest study that can be given to it.

